# A Pilot Quality Improvement Project to Reduce Intraoperative MRI Hypothermia in Neurosurgical Patients

**DOI:** 10.1097/pq9.0000000000000531

**Published:** 2022-03-30

**Authors:** Becky J. Wong, Asheen Rama, Thomas J. Caruso, Charles K. Lee, Ellen Wang, Michael Chen

**Affiliations:** From the 1 Department of Anesthesiology Perioperative and Pain Medicine, Division of Pediatric Anesthesia, Stanford University School of Medicine, Stanford, Calif.; 2 Stanford School of Medicine, Stanford, Calif.

## Abstract

Intraoperative hypothermia increases patient morbidity, including bleeding and infection risk. Neurosurgical intraoperative magnetic resonance imaging (iMRI) can lead to hypothermia from patient exposure and low ambient temperature in the MRI suite. This quality improvement project aimed to reduce the risk of hypothermia during pediatric neurosurgery laser ablation procedures with iMRI. The primary aim was to increase the mean lowest core temperature in pediatric patients with epilepsy during iMRI procedures by 1 °C from a baseline mean lowest core temperature of 34.2 ± 1.2 °C within 10 months and sustain for 10 months.

**Methods:**

This report is a single-institution quality improvement project from March 2019 to June 2021, with 21 patients treated at a pediatric hospital. After identifying key drivers, temperature-warming interventions were instituted to decrease hypothermia among patients undergoing iMRI during neurosurgery procedures. A multidisciplinary team of physicians, nurses, and MRI technologists convened for huddles before each case. Interventions included prewarmed operating rooms (ORs), blanket coverings, MRI table and room; forced-air blanket warming, temperature monitoring in the OR and iMRI environments; and the MRI fan turned off.

**Results:**

Data were analyzed for five patients before and nine patients after the institution of the temperature-warming elements. The sustainment period included 15 patients. The mean lowest intraoperative temperature rose from 34.2 ± 1.3 °C in the preintervention period to 35.5 ± 0.6 °C in sustainment (*P =* 0.004).

**Conclusion:**

Hybrid OR and MRI procedures increase hypothermia risk, which increases patient morbidity. Implementation of a multidisciplinary, multi-item strategy for patient warming mitigates the risk.

## INTRODUCTION

Perioperative hypothermia is a core temperature less than 36 °C. Hypothermia impairs intrinsic coagulation, extends postoperative recovery, and contributes to increased risk of surgical site infections.^1,2^ General anesthesia (GA) decreases heat production and inhibits the patient’s peripheral vasoconstriction resulting in a significant redistribution of hypothermia. Heat is lost to the environment through radiation, convection, conduction, and evaporation. Compared to adults, pediatric patients have a higher respiratory rate and thus lose more metabolic heat through nonheated and nonhumidified mechanical ventilation.^3^ Pediatric patients tend to cool due to less body fat and greater body surface area per weight than adults. Typically, air warming,^4^ full-body draping, and room temperature control maintain optimal thermoregulation during pediatric neurosurgery.^3^

The addition of magnetic resonance imaging (MRI) to neurosurgical procedures, referred to as intraoperative MRI (iMRI), improves the localization of seizure foci. Children with certain types of pharmaceutical-resistant epilepsy are candidates for surgical treatment with iMRI. During this procedure, after a small craniotomy, a laser catheter is placed within the epileptogenic foci, and iMRI gives surgeons information on the degree of laser ablation in time. However, patients often become hypothermic during pediatric MRIs due to the lack of nonferromagnetic forced-air warming devices and MRI machinery performance requiring cold room temperatures of 18 ± 3°C.^5,6^ Thus, despite the improved surgical feedback from iMRIs, the introduction of iMRIs to pediatric neurosurgery results in a greater risk of intraoperative hypothermia.

Given the prevalence of pediatric hypothermia during nonoperative MRIs, improvement projects aimed at reducing hypothermia in infants emphasized utilizing warming devices during anesthesia induction, MRI cooling fan stopped during imaging, and standardized MRI protocols to shorten scans.^5,7^ There are limited data on intraoperative prevention of hypothermia during iMRIs. A short case series of 10 iMRI patients for epilepsy treatment reported the increased prevalence of hypothermia, and one patient remained intubated postoperatively due to residual neuromuscular blockade and hypothermia.^8^ After initiating iMRI at our institution, patients commonly experienced hypothermia during and after the iMRI portion of the neurosurgical procedure.

Given the hypothermia experienced by patients at our institution who undergo iMRI, coupled with results from previously reported nonoperative MRI hypothermia improvement projects, we sought to reduce the risk of hypothermia in pediatric neurosurgical patients undergoing iMRI. Our institution utilizes a lean methodology improvement framework,^9^ relying on a microsystem culture, a small group of people who work together regularly to provide care. The project relied on a multidisciplinary team involvement, executive sponsorship, and A3 project planning, which is a one-page report to guide users through a systematic problem-solving process.^10^

This project aimed to reduce the incidence of intraoperative hypothermia during MRI-guided neuroablation procedures for patients with epilepsy. The primary aim was to increase the mean lowest core temperature of pediatric patients with epilepsy during iMRI procedures by 1 °C from baseline mean lowest core temperature of 34.2 ± 1.2 °C by August 30, 2020, which is 10 months since the initiation of the intervention on November 1, 2019. Therefore, the sustainment period was for 10 months until June 30, 2021. Secondary aims included exploring provider compliance with the proposed interventions and balancing measures of timeliness of patient readiness for their iMRI and total time under anesthesia. We chose the goal of a 1 °C temperature increase because of the limitations of the MRI environment prohibiting traditional forced-air warmers, coupled with the desire to create a realistic SMART goal. Forced-air warmers can conceivably warm patients up to 2.5 °C/h, but without that, passive measures such as blankets during the MRI can at best warm about 0.5 °C/h, and prewarming a bed and room also prevents about 0.4 °C drift.^11^ The 1 °C was a realistic and achievable SMART goal given our potential countermeasures. Furthermore, the goal 1 °C increases the mean lowest core temperature to above 35 °C, a threshold that is considered more severe hypothermia.^12,13^

## METHODS

### Context

The quality improvement project was conducted at a 361-bed, freestanding academic children’s hospital in Northern California. Two neurosurgical operating suites directly access a single intraoperative 3T MRI scanner (GE750W, General Electric, Boston, Mass.).^14^ The core staff involved in surgeries that include iMRI are specialty-trained for the environment. They include seven pediatric neuroanesthesiologists, two neurosurgeons, six operating room (OR) nurses, four surgical technicians, seven MRI nurses, and five MRI technologists. A multidisciplinary improvement team launched the interventions in November 2019 and completed the project in June 2021.

To control surgical confounders such as heterogeneous positioning and surgical incision sites, we included ambulatory children, 2 through 18 years of age, with epilepsy, undergoing laser ablation procedures requiring iMRI. Exclusion criteria were children who did not receive laser ablations, inpatient children before surgery, and those with pre-existing thermoregulatory disorders, such as pituitary dysfunction.

### Intervention

Prompted by the recognition of hypothermia after iMRI, a multidisciplinary team of pediatric anesthesiologists, neurosurgeons, OR nurses, MRI nurses, MRI technologists, and quality improvement specialists convened to define the current state and problem. Given the prevalence of laser ablation procedures and procedural homogeneity, the team decided to focus their improvement efforts on neurosurgical patients with epilepsy for laser ablation with iMRI to allow rapid adoption. In addition, the team instituted prospective temperature collection on a statistical process control chart and reviewed local and national guidelines.

**Fig. 1.**
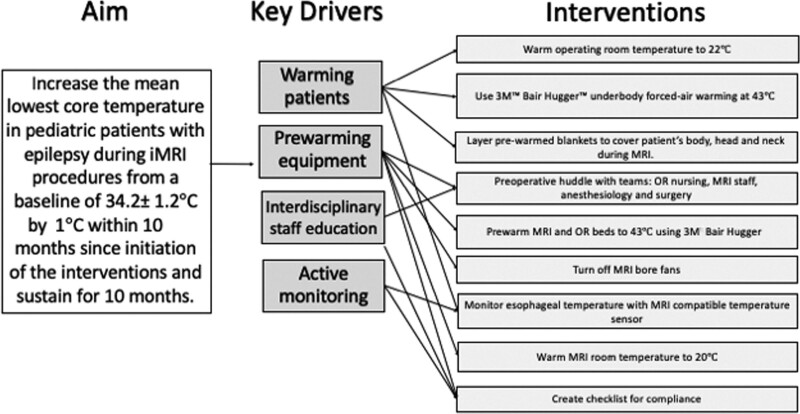
Key driver diagram. The team first identified four drivers and subsequently developed and executed nine interventions to reduce the incidence and degree of intraoperative hypothermia during MRI-guided neuro ablation procedures.

**Fig. 2.**
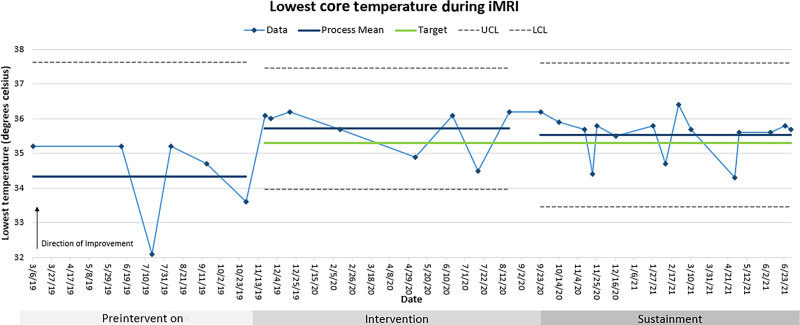
Statistical process control chart of lowest core temperature during MRI-guided neuroablation procedures. The mean lowest intraoperative core temperature rose from 34.2 ± 1.3 °C preintervention to 35.5 ± 0.6 °C during the sustainment period.

Current state analysis revealed that the team utilized Bair Hugger (3M, Maplewood, Minn.) forced-air warming devices and ambient temperature adjustments in the OR and blankets during the iMRI to maintain normothermia. The temperature was measured with an MRI-compatible esophageal thermometer (Expression MR400 Philips Invivo Monitor). The team used key drivers to guide intervention development (Fig. 1). The key drivers included active patient warming, active equipment warming, optimal interdisciplinary communication, and continuous temperature monitoring. Data collection revealed the mean lowest core temperature during the iMRI to be 34.2 ± 1.3 °C (Fig. 2). The team implemented nine interventions, including initiating a multidisciplinary preoperative huddle, utilizing a checklist for temperature settings and prewarming of equipment, prewarming of MRI and OR beds with forced-air blankets warmed to 43 °C for a minimum of 30 minutes before use, turning off MRI bore fans, increasing the MRI room temperature to 20 °C, the highest manufacturer-recommended, maintaining the OR temperature at 22 °C, utilization of under-body forced-air warming to 43 °C during the surgery, utilization of prewarmed blankets to cover fully patient’s body during the iMRI, utilization of an MRI-compatible thermometer to monitor esophageal temperature during the iMRI, and prewarming the patient to a goal of 38 °C during the operative portion in anticipation of the iMRI (Fig. 1).

The team considered a staggered approach to implementing these interventions but decided to bundle them into one, well-publicized launch in November 2019. After email correspondences, multiple team huddles, and support from executive leadership at perioperative quality improvement meetings, the team initiated the elements.

### Measures

The primary outcome was an improvement in the mean lowest core temperature during iMRI of 1 °C increase within 10 months. This change was measured with the MRI-compatible esophageal thermometer. In addition, electronic medical record (EMR) review provided minute to minute temperature recordings, and the team recorded the lowest one during the iMRI.

The secondary outcome was intervention compliance. Because neuroanesthesiologists initiated this project and given their operative role, they were responsible for leading the huddles and ensuring compliance. The improvement team reviewed intraoperative EMR data and administered surveys to neuroanesthesiologists and nursing staff to determine intervention compliance.

Given the number of proposed changes requiring preparation of the iMRI, including prewarming the iMRI room and bed, turning off the fans, setting up MRI-compatible temperature monitoring, and careful application of warm blankets without contaminating the surgical field, timeliness for starting the iMRI and duration of anesthesia were investigated as balancing measures. EMR time stamps and changes in ventilation documentation (switching from ventilation via anesthesia machine to hand ventilation during patient transport into MRI) indicated the duration of preparation time required for patients to enter the MRI scanner.

### Analysis

The improvement team used a statistical process control chart to measure the lowest core temperature during the iMRI. Centerlines (CLs) represent the mean temperature in the preintervention, intervention, and sustainment periods. There are three SDs for upper and lower control limits. CL breaks due to special cause variation were attributed to nonrandom conditions.^15^ The team collected and analyzed data using Microsoft Excel (Redmond, Wash.) and performed statistical analyses using GraphPad Prism version 9.01 (GraphPad Software, La Jolla, Calif.). The team used a *t* test to determine group temperature differences before and after the implementation of the interventions. In addition, the team used Fisher’s exact and Chi-squared tests to compare categorical variables. The team considered *P* values of <0.05 to be significant.

### Ethical Considerations

The Stanford IRB review board approved a waiver for this quality improvement project.

## RESULTS

### Demographics

Throughout the project period, the team collected data from 29 patients. There were no significant differences in patient age, weight, gender, and race between the two periods (Table 1). More patients identified as Hispanic in the preintervention period compared to the sustainment period (*P =* 0.004). The team also collected procedure characteristics (Table 2). Comparing the periods, we found no statistically significant differences in duration under GA, duration in MRI, or duration under GA after MRI.

**Table 1.** Patient Demographics

Parameter	Preintervention (n = 5)	Intervention (n = 9)	Sustainment (n = 15)	*P*
Age (y)	9.6 (± 8.6)	9.1 (± 5.2)	5.1 (± 5.2)	0.662
Weight (kg)	31.1 (± 14.6)	33.2 (± 14.7)	38.02 (± 23.8)	0.802
Gender				
Female	2	4	5	>0.999
Male	3	5	10	
Ethnicity*				
Hispanic	5	2	3	0.004
Not Hispanic	0	7	12	
Race				
White	2	4	9	0.326
Asian	0	3	2	
Other	3	2	4	

Significantly more patients identified as Hispanic in the preintervention group compared to in the sustainment group, but there were no statistically significant differences in age, weight, gender, and race between the two groups.

Age and weight are presented as mean ± SD. *P* calculated for preintervention and sustainment groups.

*P < 0.05.

**Table 2.** Case Characteristics

Parameter	PreIntervention (n = 5)	Intervention (n = 9)	Sustainment (n = 15)	*P*
Duration (min)				
Duration under GA	504 ± 172	533 ± 88	466 ± 111	0.575
Duration under GA before MRI*	84 ± 67	249 ± 92	169.3 ± 50	0.007
Duration of MRI preparation	19.2 ± 4.3	20.4 ± 4.2	16.4 ± 4.4	0.231
Duration in MRI	309 ± 113	245 ± 69	237.1 ± 73.0	0.113
Duration under GA after MRI	125 ± 200	38 ± 21	47.1 ± 37.6	0.151
Temperature (°C)				
Preoperative temperature	36.3 ± 0.6	36.6 ± 0.2	36.5 ± 0.2	0.237
Initial OR temperature*	35.0 ± 1.6	36.1 ± 0.6	36.2 ± 0.6	0.023
Lowest intraoperative temperature*	34.2 ± 1.3	35.7 ± 0.6	35.5 ± 0.6	0.004
Highest intraoperative temperature	36.5 ± 1.2	37.8 ± 0.7	37.0 ± 1.0	0.327
Temperature at the end of operation	36.3 ± 1.3	36.4 ± 0.7	36.3 ± 1.0	0.935
Postoperative temperature	36.7 ± 0.6	36.5 ± 0.2	36.7 ± 0.3	0.950
Differences in preoperative and lowest temperature*	2.1 ± 1.6	0.9 ± 0.7	0.9 ± 0.6	0.029

The sustainment group had a longer mean duration under GA before MRI, higher mean initial OR temperature and mean lowest intraoperative temperature, and smaller mean difference in preoperative and lowest intraoperative temperature than the preintervention group, at statistically significant levels.

Data are presented as mean ± SD. *P* calculated for preintervention and sustainment groups.

*P < 0.05.

### Intraoperative Core Temperature

The improvement team collected the mean lowest intraoperative temperatures from five patients before intervention adoption, nine patients after initiating the elements, and fifteen patients during sustainment (Table 2; Fig. 2). Two patients before the intervention had very low intraoperative temperatures of 32–33°C, despite experienced OR and MRI personnel. The mean lowest temperature of the patients before the intervention was 34.2 ± 1.3 °C. After implementation during sustainment, the mean lowest core temperature was 35.5 ± 0.6 °C (*P =* 0.004; Table 2; Fig. 2). Thus, the average decrease from preoperative temperature to lowest intraoperative temperature was reduced to 0.9 ± 0.6 °C in the sustainment period from 2.1 ± 1.6 °C preintervention (*P =* 0.029; Table 2).

The mean temperature at the end of surgery was similar between the two periods (36.3 ±1.3°C preintervention versus 36.3 ±1.0°C sustainment, *P =* 0.935 Table 2). However, one patient in the preintervention period had a temperature too low (32.2°C) for extubation. As a result, the care team spent an additional 69 minutes warming the patient to 35.5°C before extubation. The mean postoperative temperatures measured in the Pediatric Intensive Care Unit were similar between the two periods (36.7 ± 0.6°C preintervention versus 36.7 ± 0.3°C sustainment, *P =* 0.950; Table 2).

### Compliance of Interventions

The mean compliance after intervention adoption was 89% in the intervention period and 87% in the sustainment period per EMR audit and compliance survey. The most commonly neglected interventions in the intervention period were bed prewarming and the use of checklists with compliance rates of 56% for both (Table 3). In addition, there was decreased compliance with turning off the MRI bore fans and increasing MRI room temperature in the sustainment.

**Table 3.** Compliance of Interventions

Intervention	Intervention Compliance	Sustainment Compliance
Warming of the OR	9/9 (100%)	15/15 (100%)
Turning on bair hugger for patient	9/9 (100%)	15/15 (100%)
Layering blankets on patient in MRI	9/9 (100%)	15/15 (100%)
Conducting preoperative huddle	9/9 (100%)	15/15 (100%)
Prewarming of the surgical bed	5/9 (56%)	14/15 (93%)
Turning off MRI bore fans	9/9 (100%)	9/15 (60%)
Monitoring of temperature	9/9 (100%)	15/15 (100%)
Increasing MRI room temperature	8/9 (89%)	9/15 (60%)
Using checklist	5/9 (56%)	10/15 (67%)
Mean overall compliance	89%	87%

### Balancing Measures: Time to Initiate iMRI and Average Time under GA

The average time required for MRI preparation and average time under GA was similar between periods. The duration under GA before MRI in the preintervention period was 84 ± 67 minutes and the sustainment period was 169.3 ± 50 minutes (*P* = 0.007; Table 2).

## DISCUSSION

Patients routinely become hypothermic during hybrid surgical/MRI procedures, increasing postoperative morbidity. Unfortunately, most institutes have not developed effective strategies to mitigate this known complication of intraoperative MRI despite the risks. The interventions we described are one step toward reducing this morbidity and stand as a template for other institutes to model and build upon.

In this quality improvement project assessing pediatric patients undergoing MRI-guided neuroablation procedures, implementing the temperature-warming bundle increased the patient’s mean lowest intraoperative temperature from 34.2 ± 1.3 °C to 35.7 ± 0.6 °C initially after the interventions and to 35.5 ± 0.6 °C during sustainment. The rate of compliance with interventions was 89% in the intervention period and 87% during sustainment. In addition, implementing the elements was not costly or cumbersome, as evidenced by the similar duration under GA, MRI preparation duration, and MRI duration between the preintervention and sustainment periods. Implementing the elements did not significantly increase the time needed for patient preparation and improved the reliability and consistency of intraoperative patient temperature measurements. Overall, the project effectively reduced the degree of hypothermia while having a negligible impact on timeliness.

This project offers a unique strategy to address hypothermia, specifically in combined surgical interventions with iMRI. A previous quality improvement project focused on reducing hypothermia in isolated MRI scanning settings, initially associated with hypothermia in 65% of infants.^5^ The authors implemented various strategies, including vacuum immobilizing blankets, and reported a decline of 47% in the incidence of postscan hypothermia to 18%.^5^ Although hypothermia during MRI is not optimal, it does not carry the same risks to patients who are simultaneously undergoing a surgical procedure. Another study exploring intraoperative thermoregulation reported that 90% of infants were normothermic in the NICU before surgery or MRI, but only 43% were normothermic upon return to the NICU.^7^ The results of our study are consistent. Intraoperative MRI scanning was associated with the development of hypothermia, and the proposed elements seem to mitigate the degree of hypothermia.

Several factors made it challenging to prevent hypothermia completely. First, procedural and MRI personnel did not execute all interventions for every patient. Occasionally, OR staff are reassigned to different ORs with short notice, which may hinder the application of appropriate interventions. Second, during sustainment, there was decreased compliance in some areas, including MRI bore fan cessation and increased MRI room temperature. As is common in improvement projects, this drift was anticipated, which is why we continued to measure compliance. Targeted education will be conducted to mitigate further decay. Although previous studies have explored nonsedating alternatives for pediatric patients undergoing MRI to minimize the risk of hypothermia due to the vasodilation effects of anesthesia, such alternatives are not feasible for hybrid iMRI cases.^16,17^ Last, due to the ambient temperatures required for functioning MRI equipment, it is impossible to significantly warm the MRI suite to reduce radiant heat loss from the patient into the atmosphere.

Before implementing the bundle, several challenges made monitoring and managing patients’ temperature difficult in the setting of hybrid surgical-MRI procedures. First, due to the high risk of ferrous objects entering the MRI suite, the staff focused on identifying metallic objects before the scan more than monitoring the patient’s temperature. Furthermore, hybrid surgical-MRI procedures typically involve an increased number of personnel, which may have led to a reduced sense of agency and responsibility for adverse outcomes, such as hypothermia, through the diffusion of responsibility.^18^ After implementation of elements, the team overcame these challenges resulting in improved intraoperative temperature management.

There are limitations to the project. First, to control for heterogeneity in procedures, only patients undergoing laser ablation procedures requiring iMRI were included in the study, resulting in a small sample size. Thus, there is a small number of patients in the preintervention period. However, the surgical procedure with iMRI and neurolaser ablation is very new to our institution. The neurosurgeons began performing this procedure in March 2019, so there were no earlier patients. When we noted the extreme hypothermia, we immediately implemented the QI project to prevent patient harm. As this was a new procedure for surgeons and anesthesiologists alike, continued developments in neurosurgical technique accounted for the statistically significant difference in duration under GA before MRI between the early preintervention period and nearly a year later in the sustainment period. The duration in MRI and under GA after MRI was longer preintervention than the sustainment period, but each did not reach statistical significance. Therefore, the total duration under GA was not significantly different between the preintervention and sustainment periods. We did not examine clinical outcomes related to perioperative hypothermia, such as surgical site infection, recovery time, and length of stay. Many factors contribute to such outcomes, and our relatively small sample size would preclude us from conducting multivariable regression analysis. Second, the nonrandomized design of the project prevents us from ascertaining the causal relationship between the bundle implementation and the decrease in the incidence of hypothermia. We relied on the imperfect measure of staff recall to implement interventions such as turning off MRI bore fans and increasing MRI room temperature. Despite these constraints, the study provides effective and practical measures to address hypothermia in a unique iMRI setting. Last, there may have been uncontrolled confounders that led to the results demonstrated by the product that the study team did not measure.

## CONCLUSIONS

Hypothermia during iMRI poses a unique challenge due to limited MRI-compatible warming devices and long intraoperative MRI scans. The project reports a reduction in the occurrences of hypothermia for pediatric patients undergoing iMRI as part of the laser ablation procedures through the implementation of temperature-warming elements. Future projects may examine more detailed patient characteristics such as patient body surface area to body mass ratio and the amounts and types of anesthetic medication that can all affect thermal loss. Given the simplicity, timeliness, and low cost, the warming elements have been generalized to other iMRI procedures at our institution. Further studies investigating its efficacy in preventing or reducing complications related to intraoperative hypothermia could lead to broader clinical integration and improved pediatric perioperative care.

## ACKNOWLEDGMENTS

We would like to acknowledge the neuro-anesthesiology team, and the MRI and OR staff for changing their standard work to improve temperature regulation in our patients.

Presented in the American Society of Anesthesiology Annual Meeting 2020 (virtual) QI ePoster, October 2020.

## DISCLOSURE

The authors have no financial interest to declare in relation to the content of this article.
